# Multisite verification of the accuracy of a multi-gene next generation sequencing panel for detection of mutations and copy number alterations in solid tumours

**DOI:** 10.1371/journal.pone.0258188

**Published:** 2021-10-01

**Authors:** John Bartlett, Yutaka Amemiya, Heleen Arts, Jane Bayani, Barry Eng, Daria Grafodatskaya, Suzanne Kamel Reid, Mathieu Lariviere, Bryan Lo, Rebecca McClure, Vinay Mittal, Bekim Sadikovic, Seth Sadis, Arun Seth, Jeff Smith, Xiao Zhang, Harriet Feilotter

**Affiliations:** 1 Diagnostic Development, Ontario Institute for Cancer Research, Toronto, Ontario, Canada; 2 Laboratory Medicine and Pathobiology, University of Toronto, Toronto, Ontario, Canada; 3 Edinburgh Cancer Research Centre, Edinburgh, United Kingdom; 4 SRI Genomics Laboratory and Department of Laboratory Medicine and Molecular Diagnostics, Sunnybrook Health Sciences Centre, University of Toronto, Ontario, Canada; 5 Department of Pathology and Molecular Medicine, McMaster University, Hamilton, Ontario, Canada; 6 Hamilton Regional Laboratory Medicine Program, Hamilton Health Sciences, Hamilton, Ontario, Canada; 7 Department of Clinical Laboratory Genetics, The University Health Network, Toronto, Ontario, Canada; 8 Department of Laboratory Medicine and Pathobiology, University of Toronto, Toronto, Ontario, Canada; 9 Thermo Fisher Scientific, South San Francisco, CA, United States of America; 10 Dept of Pathology and Laboratory Medicine, The Ottawa Hospital, Ottawa, Ontario, Canada; 11 Health Sciences North/Horizon Sante-Nord, Sudbury, Ontario, Canada; 12 Molecular Diagnostics Laboratoroy, Victoria Hospital, London Health Sciences Centre, London, Ontario, Canada; 13 Department of Pathology and Laboratory Medicine, Western University, London, Ontario, Canada; 14 Department of Pathology and Molecular Medicine, Queen’s University, Kingston, Ontario, Canada; 15 Laboratory Genetics, Kingston Health Sciences Center, Kingston Ontario, Canada; CNR, ITALY

## Abstract

Molecular variants including single nucleotide variants (SNVs), copy number variants (CNVs) and fusions can be detected in the clinical setting using deep targeted sequencing. These assays support low limits of detection using little genomic input material. They are gaining in popularity in clinical laboratories, where sample volumes are limited, and low variant allele fractions may be present. However, data on reproducibility between laboratories is limited. Using a ring study, we evaluated the performance of 7 Ontario laboratories using targeted sequencing panels. All laboratories analysed a series of control and clinical samples for SNVs/CNVs and gene fusions. High concordance was observed across laboratories for measured CNVs and SNVs. Over 97% of SNV calls in clinical samples were detected by all laboratories. Whilst only a single CNV was detected in the clinical samples tested, all laboratories were able to reproducibly report both the variant and copy number. Concordance for information derived from RNA was lower than observed for DNA, due largely to decreased quality metrics associated with the RNA components of the assay, suggesting that the RNA portions of comprehensive NGS assays may be more vulnerable to variations in approach and workflow. Overall the results of this study support the use of the OFA for targeted sequencing for testing of clinical samples and suggest specific internal quality metrics that can be reliable indicators of assay failure. While we believe this evidence can be interpreted to support deep targeted sequencing in general, additional studies should be performed to confirm this.

## Introduction

The value of specific gene alterations to match individuals with cancer to molecularly targeted agents is now clear in many tumour types [1–18]. While current requirements for detection of somatic variants remain limited in specific jurisdictions such as Ontario, this is likely to change significantly as increased numbers of targeted therapies for both solid and hematologic tumours are approved by the FDA and Health Canada. Thus, it is expected that the need for broader mutation profiling for tumours will expand rapidly over the next several years.

Increasingly, broad genomic profiling, using whole genome or exome sequencing and whole transcriptome sequencing, is used in clinical trials of metastatic cancer to attempt to match patients to targeted therapeutics based on genomic features of their tumours [19–23]. Many of these trials highlight the complexity of delivering broad genomic analyses from small diagnostic samples in a timely fashion. To address this challenge a number of small and large targeted sequencing panels have been developed which cover key molecular alterations across multiple cancer types (e.g. MSK-IMPACT, Foundation One, TruSight series, Oncomine series). Among these, the Oncomine Focus (OFA) and Oncomine Comprehensive v3 (OCAv3) assays have been adopted in a number of clinical diagnostic laboratories globally. Notably, the OCAv3 is the assay of choice in the on-going Adult and Pediatric NCI- Molecular Analysis for Therapy Choice (MATCH) programs (NCT02465060/ NCT03155620) [24, 25], to allocate metastatic patients to novel targeted therapeutics. In Canada, OCAv3 is also being used by the CAPTUR, OCTANE [26, 27] and the Exactis Innovation [28] programs, to stratify patients with advanced cancer for treatment based on molecular alternations. The OFA is a smaller and more economical version of the OCAv3, and, like OCAv3, has the advantage of being appropriate for small amounts of DNA/RNA extracted from formalin fixed paraffin embedded (FFPE) diagnostic samples. Both OCAv3 and OFA can be readily adopted in routine clinical settings combining deep coverage of key genes using next generation sequencing methodology and a curated informatics reporting pipeline. Critical, however, to the implementation and subsequent clinical validation of any assay in the routine clinical setting is the ability to rely on accurate and reproducible results. In order to assess the accuracy and reproducibility of the OFA for detection of mutations, copy number alterations and fusions, we performed a ring study that included two research and five clinical molecular diagnostic laboratories in Ontario and compared results using a small series of solid tumour and control specimens.

## Materials and methods

### Study design and samples

The study was designed to assess the performance of the OFA across research and clinical laboratories in Ontario. Ethics approval was obtained from the Queen’s University Research Ethics Board (Study PATH-161-16). The study was divided into two phases. In phase 1, participating centers were provided with extracted nucleic acids from 3 control DNA (Horizon Quantitative Multiplex Reference Standard, Horizon *KRAS* Gene-Specific Multiplex Reference Standard and Horizon *EGFR* Gene-Specific Multiplex Reference Standard) and 1 control RNA (Horizon HD784) cell lines, as well as 9 DNA samples and 11 RNA samples extracted from anonymized solid tumours (Table 1). Nucleic acids for use in phase 1 were extracted at the Kingston Health Sciences Center. For phase 2, 6-μm sections from 8 of the tumours used in phase 1 were placed onto unbaked glass slides. A parallel H&E slide was examined by a pathologist, and marked to identify the tumour area. Images of the marked slides for each tumour were made available to each centre. Two slides were sent to each participating center. Each center received non-consecutive slides (i.e. slide 1 and slide 8, slide 2 and slide 9, etc) to minimize the probability of tumour heterogeneity being a major factor confounding the results. For phase 2, each center performed RNA and DNA extraction individually and used the extracted material as input to the OFA.

**Table 1:** List of samples used in the study.

Sample	Tissue	Phase 1		Phase 2
		DNA	RNA	Slides
**1**	**NSCLC**	**X**	**X**	**X**
**2**	**Colon**	**X**	**X**	**X**
**3**	**Melanoma**	**X**	**X**	**X**
**4**	**NSCLC**	**X**	**X**	**X**
**5**	**Melanoma**	**X**	**X**	
**6**	**Colon**	**X**	**X**	**X**
**7**	**NSCLC**	**X**	**X**	**X**
**8**	**NSCLC**	**X**	**X**	**X**
**9**	**Melanoma**	**X**	**X**	**X**
**10**	**Colon**		**X**	
**11**	**Melanoma**		**X**	
**DNA Std Ref**	**Cell line**	**X**		
***KRAS* DNA Ref**	**Cell line**	**X**		
***EGFR* DNA Ref**	**Cell line**	**X**		
**RNA Std Ref**	**Cell line**		**X**	

NSCLC: Non small cell lung cancer; DNA Std Ref: Horizon Quantitative Muliplex Reference Standard; *KRAS* DNA Ref: Horizon *KRAS* Gene-Specfic Multiplex Reference Standard; *EGFR* DNA Ref: Horizaon *EGFR* Gene-Specific Multiplex Reference Standard; RNA Ref Std: Horizon HD784.

### Participating centers

The following centers participated in the study: Kingston Health Sciences Center (Feilotter), Sunnybrook Health Sciences Centre (Seth), Ontario Institute for Cancer Research (Bartlett), Princess Margaret Hospital/UHN (Kamel Reid), Ottawa Hospital (Lo), London Health Sciences Centre (Sadikovic), Health Science North (McClure). Each center was provided a code letter (A-G) to anonymize results. All centers except Laboratory F used the OFA assay; Laboratory F used the OCAv3 assay. The centers running the OFA assay used either a Ion Torrent PGM sequencer and a 318 chip, or the Ion Torrent S5XL sequencer and a 520 chip. Laboratory F used an Ion Torrent S5XL sequencer and a 540 chip (Thermo Fisher Scientific, Waltham, MA, USA).

### DNA/RNA extraction

For phase 1, DNA was extracted in the Kingston laboratory, using the RecoverAll™ RNA/DNA extraction kit for FFPE tissue (Thermo Fisher Scientific, Waltham, MA, USA). DNA was provided to all sites at 2ng/μl, and participating laboratories were instructed to use 5 μl (10 ng) of each DNA solution for library preparation and sequencing. RNA samples were provided at 5ng/μl, and laboratories were instructed to use 2 μl (10 ng) of each for library construction and sequencing. For phase 2, each participating laboratory carried out their own extractions using methods that had been previously validated in their laboratories.

### Sequencing

Sequencing was performed following the instructions provided with the Oncomine Focus (Laboratories A, B, C, D, E and G) or Comprehensive (Laboratory F) Assay Library Preparation User Guide. The OFA covers hotspot mutations in 35 genes, CNV detection in 19 genes and gene fusions related to 23 genes (https://www.thermofisher.com/uk/en/home/clinical/preclinical-companion-diagnostic-development/oncomine-oncology/oncomine-focus-assay.html) and covers all recognized targets for gene targeted therapies. The OCAv3 assays includes these genes and others including: full exon coverage of 48 genes, hotspot mutation detection of 87 genes, CNV detection of 43 genes and fusions for 51 genes. Libraries were sequenced following instructions provided in the Ion Hi-Q Chef Kit, Ion 520–530 Kit Chef or Ion 540 Kit Chef reference guide. Instructions for standardized naming of samples from each site were provided, ensuring consistency across the study.

### Data generation and analysis

Sequencing runs were originally analyzed using Torrent Suite Software (TSS) versions 5.0–5.4 (depending on the timing of when runs were completed) to generate BAM files, which were uploaded to the Ion Reporter (IR) server. After completion of the study, all runs were re-analyzed using default IR Oncomine workflows in IR v5.10. DNA quality metrics were analyzed using a TSS coverage analysis plugin, and RNA quality metrics were investigated using attributes from the BAM file as well as the IR fusion workflow metrics. Minimum standards were specified according to standards provided with the OFA and OCAv3 assays. For OFA, at the time of the study, a minimum of 300,000 mapped reads for DNA and 5,000 mapped reads for RNA was required to pass quality control. More recently, the minimum number of mapped reads for RNA was changed to 50,000, and both of these metrics were used in analysis. For OCAv3, a minimum of 3,000,000 reads for DNA and 500,000 reads for RNA was required. At the time of the study, the minimum fragment length for OFA for DNA was 100, and for RNA 75 bases. More recently, this has been changed to 75 for DNA and 60 for RNA, and both metrics were considered in analysis. For OCAv3, the minimum length were set at 80 and 60 bases for DNA and RNA respectively. The minimum percent uniformity was set at 80% for both OFA and OCAv3. Variant summary files were created as VCF files. Three different types of variants (SNV/indel, copy number variant and fusion genes) were assessed using default calling settings.

## Results

### Quality metrics

Quality metrics, including read length, mapped reads (DNA & RNA) and uniformity of base coverage (DNA), are reported in Tables 2–6 for phase 1 and Tables 7–11 for phase 2. Samples not meeting the minimum standards set out in the Methods are flagged.

**Table 2:** Phase 1 DNA read length values.

Panel	OFA	OFA	OFA	OFA	OFA	OCAv3	OFA
Sample/Lab	A	B	C	D	E	F	G
**1**	**111**	**111**	**114**	**109**	**111**	**106**	**114**
**2**	**115**	**114**	**116**	**112**	**115**	**109**	**103**
**3**	**114**	**113**	**114**	**113**	**112**	**102**	**119**
**4**	**111**	**110**	**113**	**108**	**113**	**104**	**115**
**5**	**115**	**114**	**116**	**112**	**114**	**107**	**103**
**6**	**117**	**114**	**116**	**113**	**115**	**108**	**120**
**7**	**113**	**113**	**116**	**108**	**115**	**109**	**117**
**8**	**111**	**110**	**113**	**107**	**111**	**107**	**115**
**9**	**115**	**115**	**117**	**113**	**115**	**102**	**120**
**DNA Std Ref**	**118**	**117**	**121**	**116**	**ND**	**115**	**123**
***KRAS* DNA**	**117**	**117**	**120**	**111**	**122**	**112**	**122**
***EGFR* DNA**	**119**	**117**	**120**	**117**	**118**	**115**	**123**

Read lengths are provided for each sample in phase 1. The minimum acceptable read length for OFA is 100 base pairs (later changed to 75 base pairs), and for OCAv3 is 75 base pairs. ND: No data- sample failed this run for technical reasons.

**Table 3:** Phase 1 DNA mapped read values.

Panel	OFA	OFA	OFA	OFA	OFA	OCAv3	OFA
Sample/lab	A	B	C	D	E	F	G
**1**	817.88k	1.14M	1.00M	635.22k	1.26M	9.07M	427.08k
2	865.60k	793.22k	1.11M	516.94k	879.42k	8.45M	***181*.*60k***
3	975.25k	1.05M	839.76k	449.65k	745.41k	5.71M	514.47k
4	870.91k	1.02M	1.22M	733.76k	1.64M	7.45M	466.94k
5	889.95k	1.04M	1.09M	515.54k	989.02k	6.37M	***154*.*82k***
6	937.83k	1.12M	815.43k	531.01k	1.33M	8.69M	424.43k
7	909.77k	861.01k	1.31M	642.19k	1.62M	10.02M	624.47k
8	930.70k	1.02M	1.01M	508.22k	1.73M	5.51M	759.85k
9	891.46k	1.33M	863.18k	473.78k	744.14k	8.62M	383.61k
DNA Stnd Ref	968.70k	1.16M	501.39k	693.20k	ND	5.12M	697.74k
*KRAS* DNA	935.71k	822.90k	540.92k	432.01k	882.78k	7.05M	732.38k
*EGFR* DNA	878.53k	1.00M	849.01k	525.64k	684.11k	4.79M	816.08k

The number of mapped reads are provided for each sample in phase 1. The minimum acceptable mapped reads for OFA is 300,000, and for OCAv3 is 3,000,000. ND: no data- sample failed this run for technical reasons. Values in bold italics are flagged because they do not meet minimum requirements.

**Table 4:** Phase 1 DNA uniformity values.

PANEL	OFA	OFA	OFA	OFA	OFA	OCAv3	OFA
SAMPLE/LAB	A	B	C	D	E	F	G
1	99.08	97.47	98.38	92.8	98.67	91.14	96.37
2	99.05	99.48	96.92	96.99	98.26	96.6	***0*.*17***
3	96.78	98.23	97.83	94.84	97.27	87.7	97.82
4	98.94	97.76	97.6	95.8	97.54	95.17	98.2
5	98.89	99.41	95.74	96.06	97.96	93.84	***0*.*41***
6	98.98	98.16	97.17	95.87	98.13	96.15	99.07
7	99.1	96.13	98.21	95.66	98.09	94.7	99.09
8	99.05	97.49	98.19	94.83	97.97	94.46	99.09
9	98.87	97.81	96.54	98.39	98.58	81.66	99.77
DNA Stnd Ref	98.27	96.15	97.13	96.68	ND	97.34	98.89
*KRAS* DNA	97.63	92.96	96.21	96.33	94.94	96.18	98.24
*EGFR* DNA	98.89	96.86	96.81	97.04	97.67	97.47	98.56

Uniformity is provided as a percentage for each sample in phase 1. The minimum acceptable uniformity percentage for both assays is 80%. ND: No data- sample failed this run for technical reasons. Values in bold italics are flagged because they do not meet minimum requirements.

**Table 5:** Phase 1 RNA read length values.

PANEL	OFA	OFA	OFA	OFA	OFA	OCAv3	OFA
SAMPLE/LAB	A	B	C	D	E	F	G
1	91	81	96	83	90	71	98
2	97	***66***	96	91	86	83	118
3	101	93	99	93	97	***53***	106
4	96	87	99	85	94	85	107
5	97	89	98	94	93	70	118
6	99	***64***	95	92	98	79	104
7	85	***66***	94	***77***	94	84	98
8	87	85	90	84	87	90	94
9	98	***68***	98	96	98	91	105
10	87	***79***	89	***51***	86	80	97
11	96	***53***	101	88	94	*45*	101
RNA Ref Stnd	97	82	97	84	***67***	*57*	102

Read length is provided for each sample in phase 1. The minimum acceptable read length for RNA for OFA is 80 bases (later changed to 60 bases), and for OCAv3 is 60 bases. Values in bold italics are flagged because they do not meet the original minimum requirements. Values in bold italics underlined did not meet the newer requirements for OFA.

**Table 6:** Phase 1 RNA total mapped read values.

PANEL	OFA	OFA	OFA	OFA	OFA	OCAv3	OFA
SAMPLE/LAB	A	B	C	D	E	F	G
1	137.70k	71.86k	148.53k	55.09k	121.82k	991.89k	172.18k
2	183.97k	***8*.*86k***	147.96k	51.64k	132.93k	877.35k	113.32k
3	193.29k	140.72k	252.44k	***16*.*63k***	270.57k	***367*.*04k***	116.39k
4	209.62k	144.92k	240.52k	***34*.*21k***	189.05k	2.27M	142.59k
5	153.64k	232.99k	305.74k	74.35k	399.78k	630.37k	207.67k
6	180.33k	***33*.*36k***	201.73k	***46*.*63k***	225.27k	2.05M	80.29k
7	144.36k	***11*.*95k***	221.35k	***12*.*44k***	323.45k	1.22M	172.18k
8	177.19k	130.94k	174.96k	***46*.*86k***	305.90k	572.87k	101.18k
9	195.71k	51.93k	260.33k	82.77k	377.21k	1.85M	279.10k
10	88.68k	62.02k	***32*.*97k***	***2*.*61k***	230.66k	644.67k	***8*.*75k***
11	225.34k	5.97k	263.52k	***44*.*34k***	230.66k	***7*.*21k***	99.24k
RNA Ref Stnd	180.73k	83.53k	230.05k	***35*.*11k***	***2*.*16k***	***88*.*99k***	142.45k

Total mapped reads is provided for each sample in phase 1. The minimum number of reads for OFA is 5,000, (later changed to 50,000) and for OCAv3 is 500,000. Values in bold italics are flagged because they do not meet the original minimum requirements. Values in bold italics underlined did not meet the newer quality requirements for OFA.

**Table 7:** Phase 2 DNA read length values.

PANEL	OFA	OFA	OFA	OFA	OFA	OCAv3	OFA
SAMPLE/LAB	A	B	C	D	E	F	G
1	111	111	114	110	111	109	113
2	118	118	117	112	115	81	116
3	113	114	115	110	111	93	116
4	112	106	112	107	105	87	115
6	114	119	115	110	117	97	114
7	110	114	114	107	110	111	113
8	111	112	115	108	111	109	113
9	114	116	116	110	116	91	116

Read lengths are provided for each sample in phase 2. The minimum acceptable read length for OFA is 100 base pairs (later changed to 75 base pairs), and for OCAv3 is 75 base pairs.

**Table 8:** Phase 2 DNA mapped read values.

PANEL	OFA	OFA	OFA	OFA	OFA	OCAv3	OFA
SAMPLE/LAB	A	B	C	D	E	F	G
1	1.27M	1.61M	1.56M	1.54M	1.06M	8.35M	864.97k
2	1.47M	1.31M	1.82M	1.63M	1.15M	***1*.*43M***	1.12M
3	1.35M	1.49M	1.38M	1.63M	803.23k	***2*.*24M***	716.22k
4	1.09M	***184*.*06k***	1.55M	772.39k	1.12M	***1*.*82M***	3.41M
6	1.23M	1.60M	1.45M	1.28M	1.38M	4.83M	1.04M
7	1.36M	1.58M	1.43M	1.06M	845.89k	6.85M	673.43k
8	1.29M	1.55M	1.43M	1.73M	961.44k	6.32M	2.11M
9	1.36M	1.47M	718.58k	360.23k	1.36M	3.01M	1.29M

The number of mapped reads are provided for each sample in phase 2. The minimum acceptable mapped reads for OFA is 300,000, and for OCAv3 is 3,000,000. Values in bold italics are flagged because they do not meet minimum requirements.

**Table 9:** Phase 2 DNA uniformity values.

PANEL	OFA	OFA	OFA	OFA	OFA	OCAv3	OFA
SAMPLE/LAB	A	B	C	D	E	F	G
1	97.53	98.19	90.9	91.54	99.59	97	98.01
2	97.93	97.03	91.62	98.1	97.96	***53*.*85***	98.49
3	97.26	93.19	95.56	96.36	91.8	***68*.*45***	93.83
4	96.78	***69*.*22***	97.54	94.29	91.15	***61*.*36***	97.44
6	96.97	97.69	96.49	98.1	96.19	86.03	97.73
7	97.54	97.66	96.92	96.12	85.93	96.65	95.2
8	98.56	98.35	92.14	91.08	98.77	96.71	97.26
9	96.04	96.12	94.1	96.87	90.33	***68*.*03***	90.7

Uniformity is provided as a percentage for each sample in phase 2. The minimum acceptable uniformity percentage for both assays is 80%. Values in bold italics are flagged because they do not meet minimum requirements.

**Table 10:** Phase 2 RNA read length values.

PANEL	OFA	OFA	OFA	OFA	OFA	OCAv3	OFA
SAMPLE/LAB	A	B	C	D	E	F	G
1	90	***54***	92	***75***	88	***34***	95
2	100	***62***	101	***72***	99	***48***	92
3	100	***53***	101	***50***	91	63	100
4	98	***52***	95	89	82	72	98
6	98	***43***	100	81	98	***37***	101
7	95	83	94	***77***	89	***50***	93
8	91	***53***	91	***65***	92	***58***	92
9	100	***48***	102	***64***	98	97	102

Read length is provided for each sample in phase 2. The minimum acceptable read length for RNA for OFA is 80 bases (later changed to 60 bases), and for OCAv3 is 60 bases. Values in bold italics are flagged because they do not meet the original minimum requirements. Values in underlined bold italics do not meet the updated requirements.

**Table 11:** Phase 2 RNA total mapped read values.

PANEL	OFA	OFA	OFA	OFA	OFA	OCAv3	OFA
SAMPLE/LAB	A	B	C	D	E	F	G
1	285.9k	***3*.*9k***	164.5k	133.7k	166.2k	***189k***	182.9k
2	422.8k	52.4k	302.5k	81.9k	302.1k	***15k***	191.5k
3	378.0k	***28*.*5k***	268.5k	***39*.*5k***	221.8k	***92*.*6k***	217.6k
4	310.8k	***13*.*3k***	366.2k	1.8M	30.1k	***5*.*0k***	798.5k
6	277.7k	***8*.*5k***	276.6k	***23*.*1k***	300.9k	***3*.*2k***	249.0k
7	252.2k	162.6k	345.5k	***42*.*6k***	221.8k	***22*.*8k***	174.8k
8	235.2k	***7*.*0k***	228.6k	***23*.*8k***	237.0k	***425k***	343.3k
9	321.6k	***26*.*5k***	351.1k	70.4k	337.8k	***185*.*9k***	137.2k

Total mapped reads is provided for each sample in phase 2. The minimum number of reads for OFA is 5,000 (later changed to 50,000), and for OCAv3 is 500,000. Values in bold italics are flagged because they do not meet the original minimum requirements. Values in underlined bold italics did not meet the updated requirements for OFA.

### Phase 1

One sample (the DNA standard, Laboratory E) failed for technical reasons (not sequencing related) and results for this sample were not reported. No sample failed either the original or the updated read length metric (Table 2), Two samples (Samples 2 and 5) failed both the minimum mapped read requirement (Table 3) and the uniformity percentage for DNA (Table 4) in Laboratory G. Twelve samples (including 2 reference samples) failed the original RNA read length metric (Table 5), including 6 from Laboratory B, although this was reduced to 5 failed samples using the newer minimum read length requirement of 60 bases. Three samples failed the OCAv3 mapped read value (Table 6). For OFA, the RNA reference failed the original mapped read requirement in Laboratory E. Using the updated metric of minimum of 50,000 mapped RNA reads, substantially higher numbers of failures were seen, in particular from laboratories B, and D.

### Phase 2

In phase 2, no samples failed for the DNA read length values (Table 7). One sample failed for number of DNA mapped reads for OFA, and 3 samples failed OCAv3 for this metric (Table 8). The same samples, plus one additional OCAv3 sample failed the uniformity metric (Table 9). For RNA, laboratories B, D and F saw most samples fail using the original read length metric, with fewer failures (mostly in laboratory B) using the new metric (Table 10). However, using the newer metric for required minimum number of RNA mapped reads, laboratories B and D saw multiple failures across samples, while LaboratoryF was unsuccessful with the RNA runs for all samples using OCAv3.

Data from flagged samples was not considered in the following analyses unless otherwise specified.

### Control DNA/RNA samples

Table 12 shows the data from the three DNA control samples (Standard 1: Quantitative Multiplex Reference Standard FFPE; Standard 2: *EGFR* Gene-Specific Multiplex Reference Standard 5% FFPE; Standard 3: *KRAS* Gene-Specific Multiplex Reference Standard FFPE, Horizon Discovery, Waterbeach, UK). All variants were regarded as detected if expected variant allele fractions (VAFs) were greater than or equal to 5%. All variants above 5% were detected by all laboratories, apart from a single failed run (laboratory E) for DNA standard 1. Results using the RNA reference control (Standard 4: HD796 (formerly HD 784) reference from Horizon Discovery) are shown in Table 13. Five of the laboratories detected the majority of fusions, although laboratories E and F did not detect any fusions.

**Table 12:** Variant allele fractions for DNA control specimens across the ring study laboratories.

				PARTICIPATING LABS						
	GENE	AA Change	Expected (%)	A	B	C	D	E	F	G
DNA Std #1	*BRAF*	V600E	11	12	12	11	12	ND	11	11
	*KIT*	D816V	10	10	10	11	10	ND	10	9
	*EGFR*	E746_A750del	2	NC	NC	NC	NC	ND	NC	NC
	*EGFR*	L858R	3	4	NC	NC	NC	ND	NC	NC
	*EGFR*	T790M	1	NC	NC	NC	NC	ND	NC	NC
	*EGFR*	G719S	25	25	23	26	26	ND	23	26
	*KRAS*	G13D	15	13	14	16	17	ND	15	16
	*KRAS*	G12D	6	8	7	7	8	ND	6	6
	*NRAS*	Q61K	13	11	11	12	13	ND	9	11
	*PIK3CA*	H1047R	18	18	18	18	20	ND	23	19
	*PIK3CA*	E545K	9	9	9	8	8	ND	8	7
DNA Std #2	*EGFR*	L861Q	5	4	5	4	5	5	6	4
	*EGFR*	E746_A750del	5	5	6	4	4	6	4	4
	*EGFR*	L858R	5	5	6	5	8	7	5	6
	*EGFR*	T790M	5	6	7	6	5	6	5	6
	*EGFR*	G719S	5	5	5	4	5	5	5	6
DNA Std #3	*KRAS*	G12D	5	5	5	4	4	4	4	6
	*KRAS*	G13D	5	5	5	5	5	5	5	4
	*KRAS*	Q61H	5	4	5	5	5	5	5	5
	*KRAS*	A146T	5	5	5	6	7	6	4	5
	*NRAS*	G12V	5	6	5	5	4	5	4	5
	*NRAS*	Q61K	5	6	6	4	6	6	5	4

**Table 13:** Detection of fusions from the RNA control material across the ring study laboratories.

		PARTICIPATING LABS- Absolute counts (%)						
Fusion Gene A	Fusion Gene B	A	B	C	D	E	F	G
*EML4* (13)	*ALK* (20)	5870(3.25)	59(0.07)	4622(2.01)	228(0.65)	NC	NC	2984(2.09)
*CCDC6* (1)	*RET* (12)	8094(4.48)	53(0.06)	1945(0.85)	237(0.68)	NC	NC	4334(3.04)
*SLC34A2* (4)	*ROS1* (32)	17789(9.84)	4523(5.41)	27677(12.03)	9183(26.16)	NC	NC	22353(15.69)
*SLC34A2* (4)	*ROS1* (34) Alternate form detectable	1117(0.62)	73(0.09)	1361(0.59)	244(0.69)	NC	NC	1195(0.84)

Numbers are provided as absolute counts, and as fraction of total mapped RNA reads. NC = not called.

### Variant calls from clinical specimens

#### Hotspot SNV/indel calls

The 8 solid tumour samples were assessed for the presence of variant calls. Each sample had previously been assayed using a clinically validated Ion AmpliSeq Cancer HotSpot Panel v2 assay (Thermo Fisher Scientific, Waltham, MA, USA). Results from the clinically validated assay were accepted as the “true” results for each sample, and results from the ring study were measured against these expected calls.

In phases 1 and 2, a total of 11 different gain of function hotspot variants were expected (Table 14) across 11 samples run in the 7 sequencing laboratories. For this analysis, data from all runs were included to allow investigation of which quality metrics might be critical for variant calling. Overall 143/147 potential variants were successfully called (97.3%). VAFs for all of the positively called variants were highly concordant between all laboratories for both phases (Table 14). Three calls were missed by LaboratoryG due to two phase 1 samples that failed to meet the minimum number of reads and required uniformity, suggesting a problem with the quality of the sequencing for those samples. The final missed call from LaboratoryF was for a phase 2 sample, where again the number of mapped reads and uniformity were below acceptable metrics.

**Table 14:** Variant allele fractions for the gain of function variants detected by the ring study laboratories across Phase 1 and Phase 2 for the clinical specimens.

				LAB (Phase 1 VAF/Phase 2 VAF)						
SAMPLE	GENE	VARIANT	ID	A	B	C	D	E	F	G
1	*KRAS*	p.G12V	COSM20	16/14	17/11	15/14	17/13	17/13	15/11	16/19
2	*KRAS*	p.G12V	COSM20	37/39	38/41	38/39	39/39	39/39	37/26	-/19
2	*MET*	p.T1010I	COSM707	49/48	48/49	46/49	47/49	49/46	44/-	-/49
4	*KRAS*	p.G12D	COSM521	48/40	51/34	49/43	49/37	48/40	48/39	45/42
5	*NRAS*	p.Q61K	COSM580	48/na	46/na	45/na	46/na	47/na	46/na	-/na
6	*BRAF*	p.V600E	COSM476	42/47	42/44	42/47	39/42	39/44	38/44	42/42
6	*PIK3CA*	p.H1047R	COSM775	26/29	26/29	28/30	28/27	31/28	27/30	25/32
7	*KRAS*	p.G12C	COSM516	15/23	16/17	14/23	13/17	16/20	17/15	15/27
8	*EGFR*	p.L747_T751del	COSM12369	34/30	34/21	30/30	33/29	33/30	30/31	30/30
8	*PIK3CA*	p.C420R	COSM757	32/25	31/22	31/28	32/27	35/31	31/27	34/28
9	*BRAF*	p.V600E	COSM476	60/69	61/66	62/65	61/67	60/65	57/57	62/67

na: Sample was not assessed in Phase 2.

#### Copy number variant calls

Copy number variant calls are automatically assessed using the median of the absolute values of all pairwise differences (MAPD) in the Ion Reporter Software. Table 15 shows the MAPD values for each sample across each laboratory for phases 1 and 2. Generally, copy number can be assessed if the MAPD value remains below 0.5. In phase 1, Samples 1 and 9 had values >0.5 from Laboratory F. In phase 2, Sample 4 had an MAPD value greater than 0.5 for Laboratory B, and all samples from Laboratory F with the exception of sample 7 had MAPD values > 0.5. From the clinical samples, copy number gain of the *MYC* locus was consistently identified in Sample 4, with copy gain estimates ranging from 6.76 to 11.6 across both phases (Table 16).

**Table 15:** Median of the absolute values of pairwise differences across labs.

	LABS (Phase 1/Phase 2)						
Sample	A	B	C	D	E	F	G
1	0.27/0.31	0.26/0.33	0.37/0.37	0.28/0.32	0.25/0.27	0.51/0.57	0.28/0.29
2	0.19/0.25	0.19/0.22	0.27/0.33	0.26/0.18	0.21/0.2	0.39/1.04	0.21/0.22
3	0.29/0.26	0.24/0.26	0.26/0.25	0.27/0.25	0.27/0.35	0.49/0.86	0.27/0.26
4	0.2/0.27	0.23/0.53	0.25/0.27	0.27/0.34	0.23/0.28	0.48/0.87	0.25/0.28
5	0.21	0.16	0.25	0.22	0.2	0.41	0.26
6	0.2/0.4	0.24/0.29	0.31/0.28	0.29/0.24	0.2/0.24	0.48/0.65	0.26/0.3
7	0.21/0.33	0.31/0.34	0.26/0.32	0.28/0.28	0.22/0.37	0.45/0.47	0.23/0.35
8	0.27/0.3	0.27/0.3	0.34/0.42	0.3/0.3	0.3/0.25	0.4/0.54	0.27/0.33
9	0.23/0.28	0.2/0.24	0.27/0.3	0.24/0.23	0.22/0.28	0.62/0.89	0.26/0.32

Samples for which MAPD values were >0.5 are underlined.

**Table 16:** Copy number gain for *MYC* detected in Sample 4 in Phase 1 and Phase 2 across labs.

Lab	Phase-I	Phase-II
A	9.54	9.83
B	10.1	6.76*
C	9.61	8.67
D	10.7	6.89
E	10.17	8.81
F	11.6	7.74*
G	10.39	10.72

Assessment for copy number changes was limited to genes present on the OFA assay. The asterick denotes samples where the MAPD value was >0.5.

#### RNA fusions

A *MET* exon 14 skipping call was made in 3 samples in 2 labs (Samples 2, 6 and 9 in both phase 1 and 2 in Lab A and Sample 9 in both phase 1 and 2 in Lab G. All fusion calls were below 1% of total mapped RNA reads. No materials remain for orthogonal validation of the calls.

## Discussion

Targeted next generation sequencing assays, also referred to as massively parallel sequencing assays, to identify variants in tumours have become standard practice in many clinical laboratories. In Ontario, laboratories currently offer a variety of such panels, including the commonly used Ion AmpliSeq Cancer HotSpot Panel v2 assay (Thermo Fisher Scientific) as well as the TruSight panels (Illumina). In the early days of next generation sequencing, most laboratories relied on panels that detected single nucleotide variants and small insertions or deletions. However, currently more laboratories are assessing panels designed to interrogate high-level copy number changes, as well as the expression of fusion events in an effort to conserve precious sample volumes and curb the costs and the time associated with sequential multiple testing.

We engaged in a ring study including five clinical and two research laboratories across Ontario to assess the parameters of one such assay, the Oncomine Focus Assay (Thermo Fisher Scientific). The rationale was to determine the potential strengths and weaknesses of the assay and to provide important data that could subsequently assist laboratories to validate the assay in house. Ultimately, one of the clinical laboratories involved opted to utilize a larger panel (OCAv3), which limited some of the comparisons that could be made.

It was clear that the assays in all 7 laboratories were performing at a level sufficient to reliably detect all DNA variants from control cell lines at 5% VAF or greater. Although we did not do a formal study of limit of detection, we can determine that with the mean depths achieved in these studies (>1200 reads per amplicon), both assays can reliably detect SNVs and indels present in at least 5% of the molecules interrogated. Likewise, using control materials to investigate the ability of the assays to identify fusions by way of input total RNA, laboratories generally were able to detect the fusions. Laboratories unable to detect fusions in control materials were either using the OCAv3 assay and likely were limited by the amount of RNA provided, or showed failed quality metrics for both mean read length and total mapped RNA reads for the control sample. The relationship of the low quality metrics with the lack of fusion detection confirms that the quality metrics are critical components of the assay that must be tracked and used to guide interpretation. Among the laboratories that successfully detected the fusions, we noted variability in the relative number of fusion molecules detected between laboratories. This variability persisted even when visualizing the fusion results as proportion of total mapped reads. The reason for this is not clear, but does suggest that the RNA metrics for these assays require careful scrutiny, and that clinical laboratories should ensure that they independently determine minimum standards for fusion calling, which could well be somewhat different for different fusion molecules of interest.

Using clinical formalin fixed paraffin embedded specimens, clinically relevant hotspot calls were consistent (97% of clinically relevant calls were made using orthogonal data as a standard) across all assays and on all samples tested, with highly similar VAFs, with 3 exceptions. The missed calls highlighted the importance of the quality metrics associated with each sample, as all calls that were missed were from samples where the quality indicators of mapped read numbers and uniformity would have flagged the sample as substandard quality. Clearly, the assay quality metrics highlight samples where clinically relevant calls might be missed, and should be appropriately tracked and used as indicators to repeat assays, where possible. Barring that, the presence of out of range quality metrics, in particular mapped reads and uniformity, should flag a sample to be reported as inconclusive rather than negative. However, given the extremely high accuracy of detecting actionable mutations in repeat analyses across 7 laboratories using either previously extracted or locally extracted DNA, we conclude that this assay satisfies important criteria relating to accuracy and reproducibility across multiple testing laboratories for mutation detection.

Identification of copy number variants is becoming an increasingly useful technique, as large-scale or gene-level genomic amplifications or deletions are being associated with drug response or prognosis. In this study, only a single sample was shown to have an amplification of the *MYC* locus, and the calls from all of the laboratories were consistent and reproducible. Perhaps more strikingly, the numerical copy number estimates for all of the known gain of function CNV areas represented on the OFA assay were highly concordant between the participating centres. Again, given the high consistency, and reproducibility of both calls (gain/loss/no change) and copy number estimates, we conclude this to be a highly reproducible CNV assessment platform. We are limited in our ability to comment broadly on accuracy for CNV detection because a) the gains observed were not orthogonally validated (being non-actionable at this time) and b) there being only 1 gain in the samples assessed.

The RNA findings from the clinical specimens were less robust. The smaller number of assays performed and the wider quality metrics make interpretation of this part of the assay more challenging. However, although we did not have available material to confirm the presence of *MET* exon 14 skipping events in the 4 samples where that event was detected, we were able to determine that the samples in which the skipping event was called were unlikely to carry such a biomarker. Of the 4 clinical specimens with a suggested *MET* exon14 skipping event, two were melanoma samples, one of which also carried a driver *NRAS* mutation, one was a colorectal cancer and the last was a lung cancer sample with a *KRAS* driver mutation. These tumours would be unlikely to harbour a *MET* exon 14 skipping driver mutation, suggesting that these calls could be false positives. Indeed, more recent developments with the OFA/OCAv3 assays suggests that calling this particular RNA-based biomarker requires careful calibration of the assay, ensuring that the skipping event is called with a minimum of 1000 reads [29]. The remaining fusion calls that were identified in three additional phase 2 samples are also likely to be false positives, given they were not detected by most laboratories, and never in the matched phase 1 samples. The variability seen in the RNA portion of the assays highlights again that this part of the assay is more vulnerable to laboratory handling and workflow. Of interest the quality metrics for the RNA results were not markedly different between phase 1 (where pre-extracted mRNA was shipped to laboratories) and phase II (where laboratories extracted RNA locally). This suggests that the quality issues are not related to degradation of samples during shipping. Clearly, the metrics that accompany the assays are relevant for all clinical laboratories to ensure high quality results, but the RNA aspects of the assays likely require independent assessment of lab-specific metrics to ensure consistency and to limit false calls.

Overall we demonstrate that the OFA is a highly accurate and reproducible platform, in both the clinical diagnostic and research setting, for the detection of SNVs and CNVs using low input FFPE derived DNA. We have limited data about OCAv3, given that only a single laboratory used this assay, and results from this laboratory were likely compromised by the limited amount of material that could be shared and the 2X higher input amounts needed for that assay. Since only one participating laboratory used the OCAv3 assay we cannot extend our broader findings relating to OFA to OCAv3, or indeed other assays. The major limitation of this study is the small number of samples used, which was due to the difficulty of identifying clinical specimens with sufficient material to be shared cross multiple laboratories. Despite the limited number of samples, however, the data do provide important guidance about quality metrics that should be monitored for use of both DNA and RNA as input materials for next generation sequencing assays. Overall, this study further strengthens the case for using panel-based testing for small samples with limited amounts of available diagnostic material and provides some insights into the use of quality metrics to flag compromised samples with a high risk of false negative results. It also provide important insights into the importance of standardized protocols, training and robust clinical validation prior to the use of these assays in the clinical setting. Specifically, important insights from this study suggest that a) the quality metrics tracked in the study for both DNA and RNA components are critical elements of the analytic process and should be part of any standardized approach to assessing the assays, and b) that the RNA component appears more variable, suggesting that clinical validation for each fusion requiring detection might be considered independently. As panel-based targeted sequencing becomes more widely available, studies like the one presented here may form an invaluable source of data to inform quality assurance and assessment approaches to diagnostic targeted sequencing assays.

The use of panels such as OFA or OCA in the clinical setting for patients means that even patients with very small amounts of tumour material may be able to access molecular testing to guide their clinical management. In Ontario, testing for predictive biomarkers including *EGFR* for lung adenocarcinoma, *BRAF* in malignant melanoma and *KRAS* and *BRAF* in metastatic colorectal cancer supports the use of targeted therapies for these patients. Assays such as those investigated in this study will continue to provide this critical information to patients as these indications continue to expand. The onus on the laboratories continues to be to ensure that the metrics guiding the use of these assays are well understood.
